# Towards a FISH-based karyotype of *Rosa* L. (Rosaceae)

**DOI:** 10.3897/CompCytogen.v10i4.9536

**Published:** 2016-11-04

**Authors:** Ilya V. Kirov, Katrijn Van Laere, Nadine Van Roy, Ludmila I. Khrustaleva

**Affiliations:** 1 Center of Molecular Biotechnology, Russian State Agrarian University - Moscow Timiryazev Agricultural Academy, Timiryazevskay str. 49, 127550, Moscow, Russia; 2 Department of Genetics and Biotechnology, Russian State Agrarian University - Moscow Timiryazev Agricultural Academy, Timiryazevskay str. 3, 127550, Moscow, Russia; 3 Institute for Agricultural and Fisheries Research (ILVO), Plant Sciences Unit, Applied Genetics and Breeding, Caritasstraat 39, 9090, Melle, Belgium; 4 Center of Medical Genetics, Faculty of Medicine and Health Sciences, Ghent University, De Pintelaan 185, 9000, Ghent, Belgium

**Keywords:** Cytogenetic markers, fluorescence in situ hybridization, interstitial telomeric repeat (ITR), 5S rDNA, 45S rDNA, Rosa wichurana

## Abstract

The genus *Rosa* Linnaeus, 1753 has important economic value in ornamental sector and many breeding activities are going on supported by molecular studies. However, the cytogenetic studies of rose species are scarce and mainly focused on chromosome counting and chromosome morphology-based karyotyping. Due to the small size of the chromosomes and a high frequency of polyploidy in the genus, karyotyping is very challenging for rose species and requires FISH-based cytogenetic markers to be applied. Therefore, in this work the aim is to establish a FISH-based karyotype for *Rosa wichurana* (Crépin, 1888), a rose species with several benefits for advanced molecular cytogenetic studies of genus *Rosa* (Kirov et al. 2015a). It is shown that FISH signals from 5S, 45S and an *Arabidopsis*-type telomeric repeat are distributed on five (1, 2, 4, 5 and 7) of seven chromosome pairs. In addition, it is demonstrated that the interstitial telomeric repeat sequences (ITR) are located in the centromeric regions of four chromosome pairs. Using low hybridization stringency for ITR visualization, we showed that the number of ITR signals increases four times (1–4 signals). This study is the first to propose a FISH-based *Rosa wichurana* karyotype for the reliable identification of chromosomes. The possible origin of *Rosa wichurana* ITR loci is discussed.

## Introduction

*Rosa* Linnaeus, 1753 is an economically important ornamental genus belonging to the Rosaceae. Of the approximately 200 described *Rosa* species (Wissemann and Ritz 2005), only 8 to 15 species contributed to the original germplasm of the modern rose cultivars. *Rosa* is one of the most widely cultivated ornamental plants worldwide, but few basic molecular cytogenetic studies in *Rosa* have been performed, including chromosome counts and karyotyping (Wylie 1954, Price et al. 1981, Liu and Li 1985, Subramanian 1987, Ma et al. 1997, Fernandez-Romero et al. 2001, Akasaka et al. 2002, 2003, Jian et al. 2013a, 2013b). Performing molecular cytogenetics in roses is a big challenge due to their very small genome size (the diploid genome size is 0.83 to 1.30 pg/2C, Roberts et al. 2009), small chromosomes (Kirov et al. 2014a), low mitotic index in roots and shoots, and weak root development (Ma et al. 1996). Moreover, most wild roses are polyploids (Vamosi and Dickinson 2006), ranging from diploid (2n = 2x = 14) to decaploid (2n = 10x = 70) (Roberts et al. 2009, Jian et al. 2010).

*Rosa wichurana* (Crépin, 1888) is a valuable model species for molecular cytogenetic studies in *Rosa* genus (Kirov et al. 2015b). It is a diploid species (2n = 2x = 14) with suitable apical and root meristems that can be used for chromosome preparations. *Rosa wichurana* is involved in the origin of modern rose cultivars and is one of the parental species used for the construction of several rose genetic maps (Crespel et al. 2002, Dugo et al. 2005, Shupert et al. 2007, Spiller et al. 2011, Moghaddam et al. 2012). To increase the efficiency of FISH experiments, we recently developed the “SteamDrop” protocol for the preparation of high quality chromosome slides (Kirov et al. 2014b). Using this “SteamDrop” protocol and Tyramide-FISH it was possible to physically map several single-copy genes on the mitotic and meiotic chromosomes of *Rosa wichurana* (Kirov et al. 2014a, Kirov et al. 2015a) and to anchor three linkage groups of the genetic map (Moghaddam et al. 2012) to three *Rosa wichurana* chromosomes.

Because the chromosomes are difficult to distinguish, further progress in cytogenetic mapping depends on the development of cytogenetic markers useful for chromosome identification. The conservative tandemly organized repetitive sequences 5S and 45S rRNA genes are valuable sources of cytogenetic markers, and have been used for chromosome identification in many plant species including *Rosa* species (Ma et al. 1997, Fernandez-Romero et al. 2001, Akasaka et al. 2002, 2003, Lim et al. 2005, Jian et al. 2012, Kirov et al. 2014a). Other conservative repeats, such as the *Arabidopsis*-type telomeric repeat (Fuchs et al. 1995, He et al. 2013) might be used for chromosome identification. Typically, telomeric repeats (TRs) occupy the end (telomere) of the chromosomes (Fuchs et al. 1995). However, the location of TRs on plant chromosomes is not restricted to the telomere ends and telomere-like sequences have been found in centromeric, subtelomeric and interstitial regions in several genera (Fuchs et al. 1995, Uchida et al. 2002, Tek and Jiang 2004, Mlinarec et al. 2009, Mandakova et al. 2010, Gong et al. 2012, He et al. 2013, Sousa et al. 2014). The unique position of these interstitial telomeric repeats (ITRs) on some chromosomes and their high copy number make them valuable cytogenetic markers. The position of ITR on chromosomes can also reflect ancient chromosomal rearrangement as telomeric sequences and their remnants are involved in chromosomal rearrangements via illegitimate recombination between centromeric/telomeric repeats (Murat et al. 2010) and can be associated with fragile sites of chromosomes (Grabowska-Joachimiak et al. 2015). In addition, the chromosomal location of ITR can be used to detect descending dysploidy (Sousa and Renner 2015).

Development of an effective cytogenetic marker system is an important step in answering many biological questions (Jiang and Gill 2006). FISH-based markers have shown their effectiveness and ease-to-use. The modern methods of probe labeling and the application of directly labeled oligonucleotides make FISH-based chromosome identification a robust and fast procedure (Kato et al. 2004, Fu et al. 2015, Tang et al. 2014, Cuadrado et al. 2009). Up-to-date FISH based karyotyping was established for many plant species including wheat, maize, rice, soybean, common bean and others (Cheng et al. 2001, Kato et al. 2004, Findley et al. 2010, Iwata-Otsubo et al. 2015). Cytogenetic markers are widely used to trace individual chromosomes in hybrids accelerating transferring of desirable traits from wild relatives (Szinay et al. 2010). FISH-based karyotyping is used to shed light on speciation and allopolyploid formation (Badaeva et al. 2016). And a relatively new application came with the development of a FISH-based chromosome sorting procedure, allowing individual chromosome identification, sorting and further sequencing (Giorgi et al. 2013). These and other applications clearly demonstrate the importance of having a system of cytogenetic markers enabling chromosome identification.

This study aims to explore the opportunities of ITRs, 5S and 45S rDNA as cytogenetic markers allowing to distinguish individual chromosomes of *Rosa*. FISH with 5S rDNA, 45S rDNA and the *Arabidopsis*-type telomeric repeat was performed. These FISH results were combined with chromosome morphology measurements (Kirov et al. 2014a), in order to identify all seven mitotic chromosomes of *Rosa wichurana*. In addition, we also attempted to identify pachytene bivalents by FISH using the 45S rDNA and *Arabidopsis*-type telomeric repeat probes.

## Materials and methods

### Plant material

*Rosa wichurana* plants were grown in the field. For chromosome slide preparations, cuttings were made. Rooted cuttings were transferred to terracotta stone pots and grown in the greenhouse (moderate climatic conditions, East Flanders, Belgium). To prepare mitotic chromosome slides, young meristems were harvested. For meiotic (pachytene) chromosome slides, flowers buds with a hypanthium size of 3 mm were harvested.

### Probe labeling

Plasmids containing 5S rRNA genes of rye (pSCT7, Lawrence and Appels 1986) and 45S rRNA genes of wheat (pTA71, Gerlach and Bedbrook 1979) were labeled by Digoxigenin- and Biotin- Nick Translation Mix (Roche, Germany), respectively, according to the manufacturer’s protocol. The *Arabidopsis*-type telomere repeat (CCCTAAA)_3_, labeled by TAMRA at the 5’ end (Syntol, Russia) was used.

### Chromosome preparation and fluorescence *in situ* hybridisation

Pachytene and mitotic chromosomes were prepared according to the “SteamDrop” protocol (Kirov et al. 2014b).

For FISH we used the protocol described in Heslop-Harrison et al. (1991) with some modifications. Briefly, slides were incubated overnight at 37°C. Chromosomes were pretreated with 4% paraformaldehyde in 2xSSC (pH 8.3–8.5) for 6 min and dehydrated in ethanol (70%, 90% and 100%). Hybridization mixture consisted of 50% (v/v) deionized formamide, 10% (w/v) dextran sulphate, 2xSSC, 0.25% sodium dodecyl sulphate, 2.00 ±1.00 ng/µl probe DNA. The mixture was denatured at 75°C for 5 min, placed on ice for 5 min and 60 µl was applied on each slide. Slides were denaturated at 75°C for 5 min and incubated in a humid chamber for 15–16 hours at 37°C (the common hybridization condition) or at 23–25°C (the low stringency hybridization condition). For stringency washing 0.1xSSC solution was used at 48°C (2 times 7 minutes). Biotin and digoxigenin labeled probes were detected by Streptavidin-Cy3 (Sigma-Aldrich, USA), diluted 1:200 in TNB buffer, and anti-digoxigenin-FITC (Roche, Germany), diluted 1:200 in TNB buffer, respectively.

For sequential FISH experiments, the slides were washed in the series of ethanol (70%, 90% and 100%) after the first round of FISH and then the above-mentioned FISH procedure was applied.

### Microscopy and image analysis

Images were acquired using a Zeiss AxioImager M2 fluorescence microscope (400× and 1000× magnification) equipped with an AxioCam MRm camera and Zen software (Zeiss, Belgium). Final image adjustments were performed using Photoshop (Adobe Inc., USA). Measurements of chromosome lengths and karyotyping was done in MicroMeasure version 3.2 (Reeves and Tear 2000) for at least 10 well-spread metaphases.

## Results

### FISH using Arabidopsis-type telomere repeat, 5S rDNA and 45S rDNA allows unambiguous identification of 3 *Rosa wichurana* mitotic chromosomes

**Figure 1.**
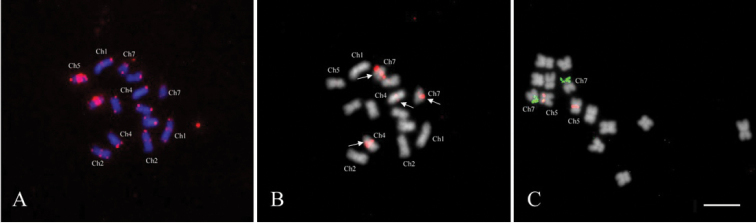
FISH on the chromosomes of *Rosa wichurana*. **A** FISH with *Arabidopsis*-type telomere probe (red) and 45S (green) under hybridization at 37°C **B** FISH with *Arabidopsis*-type telomere probe under the low hybridization stringency condition (23-25°C). Arrows indicate the major ITRs on chromosome 5 and arrowheads show the ITRs which are visible under the low hybridization stringency condition **C** The same metaphase as in 1B rehybridized with 5S rDNA under the common hybridization stringency (37°C). Arrows indicate the 5S rDNA signals. Sacale bar: 5 µm.

FISH using the common hybridisation temperature of 37°C with 45S rDNA revealed a signal on chromosome 7, while the *Arabidopsis* type telomere-based probe hybridized on chromosome 5 (Fig. 1A).

To further evaluate the value of the telomeric repeat (TR) as a cytogenetic marker, FISH was carried out at room temperature (the low hybridization temperature). We observed the *Arabidopsis*-type TR signals on all chromosome ends (Fig. 1B). Besides the telomeric signals, a bright fluorescent signal in the centromeric region on chromosome 5 and weak signals in the centromeric region on three other chromosomes 1, 2 and 7 were observed. Remarkably, the weak centromeric signals on chromosomes 1, 2 and 7 were not observed when performing a hybridization at 37°C (Fig. 1A). No ITRs were present on chromosomes 3, 4 and 6. FISH with 5S rDNA using the common hybridization temperature of 37°C showed fluorescent signals on the long arm of chromosomes 4 and 7 (Fig. 1C) but the signal frequency across the metaphases was low (20–40%).

**Figure 2.**
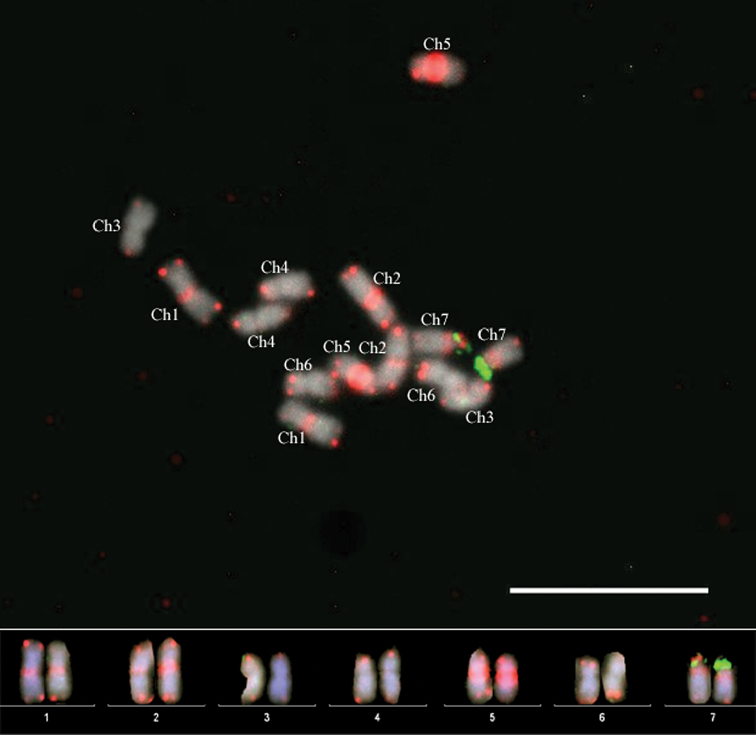
Double-color FISH under the low hybridization conditions using the *Arabidopsis*-type telomere repeat-based (red) and 45S rDNA (green) probes to *Rosa wichurana* mitotic chromosomes. Scales bar: 10 µm.

Sequential FISH at the low hybridization temperature with the *Arabidopsis*-type telomere-based probe and 5S rDNA showed co-localization of these signals on chromosome 7. We also performed double-color FISH with the *Arabidopsis*-type telomere repeat-based probe and the 45S rDNA probe under the low temperature of hybridization (Fig. 2) which confirmed the identification of four (1, 2, 5 and 7) out of seven chromosomes.

**Figure 3.**
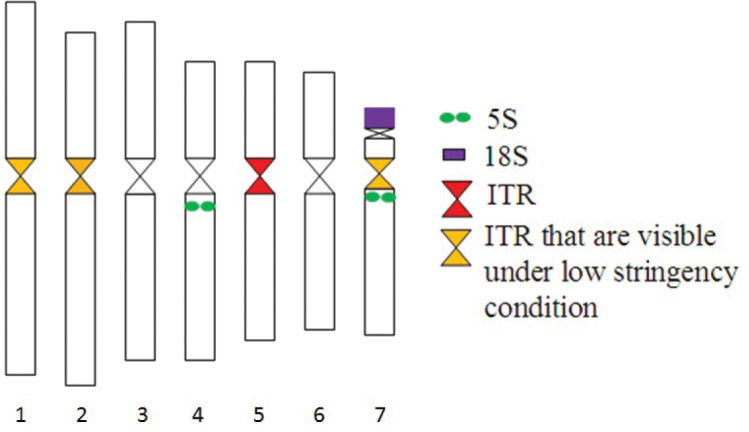
Distribution of the repetitive sequences on the mitotic *Rosa wichurana* chromosomes. ^1^ – ITR1: signals that are visible under hybridization at 37°C as well as at low temperature (23–25°C). ^2^ – ITR2: signals that are visible only under hybridization at low temperature (23–25°C).

A summary of the karyotypic features and distribution of FISH probes is given in Fig. 3. Taken together, three chromosomes (4, 5 and 7) of *Rosa wichurana* could be unambiguoulsy identified by 5S rDNA, 45S rDNA and the *Arabidopsis*-type TR using common FISH hybridisation conditions (Fig. 3).

All the other chromosomes can only be distinguished at this time based on their morphological parameters. Differentiation between chromosome 1 and 2 is possible by their centromeric indices which are 46.00 ±1.2% and 40.30 ±1.3%, respectively (Kirov et al. 2014a) and by the presence of an ITR when using FISH at low temperature hybridization conditions. Chromosomes 3 and 6 have centromeric indices on the level of 44.3 ±1.0% and 41.8 ±1.1%, respectively (Kirov et al. 2014a). However, these chromosomes still remain very difficult to distinguish from each other.

### ITRs are located on the centromere of chromosome 5

**Figure 4.**
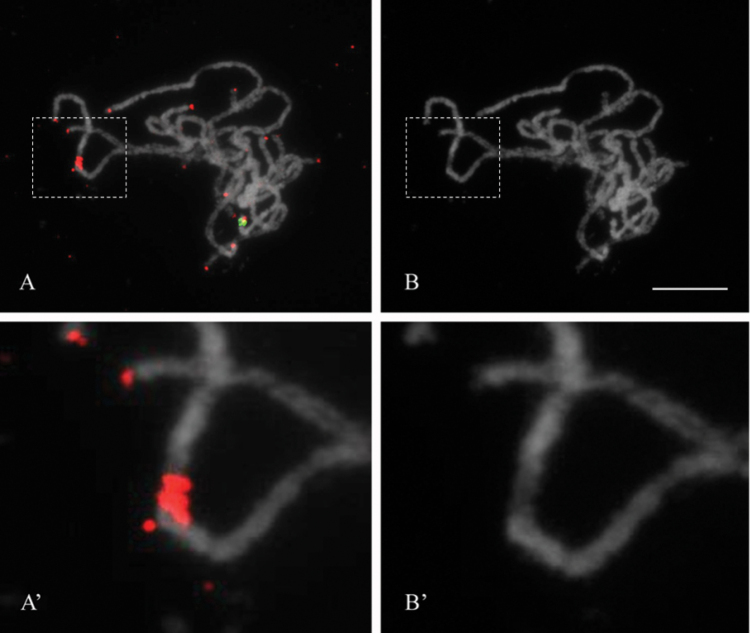
High resolution physical mapping of ITR on *Rosa wichurana* pachytene chromosomes. FISH with the *Arabidopsis*-type telomere repeat probe (red) and 45S (green). Merged (**A**) and the DAPI gray scale (**B**) pictures are shown. FISH was performed under the low hybridization stringency condition. Dotted lines show the regions that were digitally enlarged (A’ and B’). Scales bar: 5 µm.

FISH experiments with 5S rDNA, 45S rDNA, and the *Arabidopsis*-type TR on rose pachytene chromosomes provide a much higher resolution of the mapped sequences. 5S rDNA-FISH on pachytene chromosomes did not reveal any reliable signals, while FISH with the 45S rDNA probe resulted in a clear signal at the subtelomeric region of the NOR-bearing chromosome (Fig. 4). FISH with the *Arabidopsis*-type TR probe resulted in signals on all ends of pachytene chromosomes and one bright signal on the centromeric region of chromosome 5 (Fig. 4). Since centromeres of rose pachytene bivalents are clearly visible after DAPI staining as being the weakest part of the chromosomes (Kirov et al. 2015a), comparison between the DAPI stained chromosomes (Fig. 4B’) and the ITR signal positions (Fig. 4A’) revealed that the ITRs are located exactly on the centromere of chromosome 5.

## Discussion

*Rosa* mitotic and meiotic chromosomes are difficult to distinguish by common karyotype analysis (Kirov et al. 2014, Kirov et al. 2015a). The development of cytogenetic markers is necessary for individual chromosome identification and further cytogenetic studies in *Rosa*. In our study, we positively evaluated the use of the conservative tandem repeats, *Arabidopsis*-type telomere, 45S and 5S probes, as FISH-based cytogenetic chromosome markers for *Rosa wichurana*. However, the 5S rDNA probe cannot be considered as a good cytogenetic marker for *Rosa wichurana* chromosomes due to the low reliability of the FISH-signals. Application of FISH with the 5S rDNA probe to chromosome slides prepared by an alternative method (spread protocol of Pijnacker and Ferwerda (1984)) and using FAM labeled 5S oligos or a *Rosa wichurana* 5S clone as probes, did not improve FISH results (data not shown). Thus the reason for weak 5S rDNA FISH signals on *Rosa wichurana* chromosomes remains unclear. FISH with the *Arabidopsis*-type TR under low hybridization conditions (hybridization at 23-25°C instead of 37°C) provided us an additional tool for identification of *Rosa* chromosomes.

In this study, FISH with the 45S rDNA and the *Arabidopsis*-type telomere probe, reliably identified 2 (chromosome 5 and 7) of the 7 pachytene bivalents of *Rosa wichurana*. These markers will accelerate the ongoing physical mapping of pachytene chromosomes of *Rosa wichurana* as their identification by morphological parameters or specific heterochromatin patterns is impossible (Kirov et al. 2015a).

ITRs can be used to trace ancient chromosomes rearrangements such as chromosome fusions, Robertsonian translocations and duplications resulting in dysploidy (Mandakova et al. 2010, Sousa et al. 2014). However, *Rosa* species have a basic chromosome number n = 7, suggesting that no descending dysploidy, which usually results in basic chromosome number changes, has occurred. Therefore, it seems unlikely that the observed ITRs are the indications of such chromosome fusions or translocations. ITRs might also be the traces of intrachromosomal rearragements implicating telomeres (e.g., inversions and duplications) (Murat et al. 2010). In our study, the *Arabidopsis* telomere-like motif was found in centromeric repeats of *Rosa wichurana*, as is also observed in several other genera (Tek and Jiang 2004, He et al. 2013, Emadzade et al. 2014). The FISH signal from ITRs on chromosome 5 is significantly stronger than those observed in the telomeres of *Rosa wichurana* chromosomes. Thus, we hypothesize that the occurrence of ITRs in the centromeric regions of *Rosa wichurana* chromosomes is the result of insertion of *Arabidopsis* telomere-like sequence into centromeric sequence followed by massive amplification of centromeric tandem repeat(s) containing an *Arabidopsis* telomere-like motif. To check this hypothesis identification of centromeric repeats of *Rosa wichurana* should be done (Tek and Jiang 2004). The events leading to insertion of ITR sequences into centromere are unknown.

Interestingly, FISH under the low hybridization temperature – and thus low stringency – revealed more chromosomes possessing the telomeric repeat compared to FISH performed under the common hybridization temperature. This result suggest that these chromosomes (1, 2 and 7) may contain truncated or diverged telomere motifs. As a consequence for our experiments, the telomeric probe may be much more informative as cytogenetic marker when hybridized at a lower temperature than at 37°C (Fuchs et al. 1995, Tek and Jiang 2004, Sousa et al. 2014, Sousa and Renner 2015). However, the application of ITR markers under the low-hybridization stringency and simultaneous mapping of other probes (e.g. genes) can be challenging as non-specific hybridization signals may occur due to low stringency. In this case sequential FISH can be applied.

High-resolution FISH on pachytene chromosomes with the telomere probe resulted in a signal in the centromere of chromosome 5, indicating that the telomere-like motifs may be the components of the *Rosa wichurana* functional centromere as it has been shown for potato (Tek and Jiang 2004).

This is the first report describing valuable cytogenetic markers for four mitotic chromosomes and two pachytene bivalents of *Rosa wichurana*. Moreover, by combining our FISH results with the chromosome morphology measurements (Kirov et al. 2014a), all 7 mitotic chromosomes of *Rosa wichurana* could be identified. Because *Rosa wichurana* has many advantages as a model species for cytogenetic studies of the *Rosa* genus, the development of a complete set of cytogenetic markers should facilitate the physical mapping of its genome. Designing new DNA probes based on NGS data covering all chromosomes of *Rosa wichurana* is a scope for our future research. These markers will be indispensable for high-resolution physical mapping experiments (Kirov et al. 2015a) that are currently ongoing for this species.
